# Psychosocial outcomes and peer influences among multiracial adolescents in the United States

**DOI:** 10.3389/fpubh.2023.852268

**Published:** 2023-02-27

**Authors:** Stephanie A. Grilo, John S. Santelli, Constance Nathanson, Marina Catallozzi, Ana F. Abraido-Lanza, Sarah Adelman, Diana Hernández

**Affiliations:** ^1^Mailman School of Public Health, Heilbrunn Department of Population and Family Health, Columbia University, New York City, NY, United States; ^2^Mailman School of Public Health, Department of Sociomedical Sciences, Columbia University, New York City, NY, United States; ^3^Department of Pediatrics, Columbia University Medical Center, New York City, NY, United States; ^4^School of Social Work, Columbia University, New York City, NY, United States

**Keywords:** adolescents, multiracial, psychosocial, peer influence, disparities

## Abstract

**Objective:**

To examine racial and ethnic self-identification among adolescents and explore psychosocial outcomes and peer treatment for multiracial adolescents in the United States.

**Methods:**

Data are from the 2014 Child Development Supplement, a subsample of the Panel Study of Income Dynamics. Data were weighted to be nationally representative. Descriptive statistics were used to describe the population and to explore family and parent demographics. Multivariable regressions tested for differences in psychosocial outcomes and peer treatment and group behaviors for multiracial youth in comparison to their single race peers.

**Results:**

Black multiracial youth had significantly lower scores on the children's depression index compared to single race Black youth, and White multiracial youth reported significantly higher rates of peer mistreatment in comparison to White single race youth. Black multiracial and White multiracial adolescents reported similar positive and negative peer group behaviors.

**Conclusions:**

Complex patterns emerge when examining the psychosocial and peer treatment variables presented in this analysis for multiracial adolescents and their single-race peers. The findings regarding depressive symptoms and peer bullying point to signs of different relationships between multiracial groups. White multiracial adolescents report worse outcomes than their White single-race peers, but Black multiracial adolescents reporting better outcomes than their Black single-race peers.

## Introduction

### The multiracial population

The percentage of individuals in the United States who identify as multiracial has been steadily increasing. Between 2010 and 2020 the increase on the US census of those who identify as more than one race was 276%, from 9 million to 33.8 million (1). As the proportion of the population that identifies as multiracial continues to grow, assumptions are being made about the process of claiming a multiracial identity and outcomes for this population. It is critical to have a demographic understanding of the multiracial segment of the population, and to understand the many influences on this identification for young people. Adolescents do not exist in a vacuum as they are intertwined as families and peer networks, both of which impact how and why they choose certain identifications and how that identification may or may not impact or be associated with certain behaviors and/or outcomes.

It is critical to operationalize the multiple terms used when discussing race and ethnicity. Racial or ethnic identity or identity formation refers to the self-understanding of race/ethnicity, racial identification relies on others identification of an individual's race, and finally categorization refers to how an individual chooses or selects a racial/ethnic group given a closed set of categories (2). In this study adolescents were asked to self-identify and therefore we are exploring the influences on and effect of racial or ethnic identity. We are unable to explore racial identification and how adolescents are perceived by their peers because that data is not included.

### Psychosocial wellbeing

Previous research has examined mental health outcomes for multiracial youth. The public health research that exists on this topic is risk-based and focuses on mental health variables such as increased levels of anxiety, stress, and depressive symptoms as mediators between identification as multiracial and poor health and behavioral outcomes (3, 4). A study by Fisher and colleagues aimed to explore the relationship between ethnic identity and mental health outcomes for multiracial adolescents. They found that multiracial youth experienced higher levels of anxiety and depressive symptoms in comparison to their single-race peers and more symptoms of anxiety than their White peers (5). It is critical to note that the data used in the Fisher study by were collected in 2006 in a Midwestern state and were not nationally representative. Therefore, it is critical that additional research is done examining multiracial populations and mental health outcomes in more representative geographic locations. A study by Weller and colleagues examined the influence of adverse childhood events on multiracial adolescent mental health and found that household dysfunction may be one of the underlying factors that influences mental health conditions among multiracial adolescents (6). Household dysfunction may particularly impact multiracial adolescents because it may cause separation from one parent, and therefore may result in less ability of the adolescent to maintain the racial and ethnic experiences from that parent that are necessary in developing their identity (6).

### Peer influences

In addition to familial influences, peer groups also play important roles in racial and ethnic identification for multiracial adolescents and young adults (7, 8). Most of the previous research has focused on the impact of peers' race and ethnicity on multiracial adolescents' identification. Some research has explored to what extent and at what ages there is more racial fluidity among multiracial populations, and the impact of peer influences on that classification (7). Not only are peers important to examine in terms of their influence on racial and ethnic identification, but also in terms of risk behavior and involvement. A study done by Choi et al. found that multiracial youth were more likely than their single-race peers to be impacted by peer pressure (4). However, this study did not examine in-depth reasons why that influence was greater, or what behaviors their peers engaged in that were considered risky. This current paper aims to examine the perception of adolescents who identify as multiracial about treatment from their peers as well as their report of peer network behaviors. These analyses are included in this paper in order to test the hypothesis that adolescents who identify as multiracial are more likely to be influenced by negative or risky peer groups due to a need to “fit in” (4).

This study utilizes nationally representative data from both adolescents and their parents to: (1) describe the multiracial population of adolescents in a nationally representative survey in the United States; (2) explore psychosocial wellbeing and peer influences including markers for mental health and negative and positive peer influences of these adolescents.

## Methods

Data from this analysis are from the Panel Study of Income Dynamics (PSID), a large, longitudinal panel study in the United States that focuses on issues of family, income, education, health behaviors, and many additional topics. Specifically, this study uses data from the Child Development Supplement (CDS) 2014 data. The CDS is designed to be nationally representative in terms of the US population of children and families. To be eligible to participate: the family must have participated in the core PSID survey in 2013; the child must have been born between 1997 and 2013; the child belonged to the PSID sample, the child was not the household head and was not in the previous CDS study. The total sample of children that were eligible to be included in the CDS 2014 was 5,816 (9).

Families with eligible children were contacted and completed a “cover screen” which asked questions about household composition and the primary caregiver. The final number of children that data were collected from was 4,333 (77%)—the remaining were left out for a multitude of reasons including refusal, the family not being located, a language barrier, office error, or their primary care giver did not respond to multiple contact attempts. Children in the final sample ranged from ages 0–17, and the sample was roughly even between males and females. The CDS includes multiple data sources, including a primary caregiver household interview, a primary caregiver child interview, a child interview, child assessments (for those families selected), a time diary (for those families selected), a demographic file and a file mapping the data back to the larger PSID sample. For the sake of this analysis, data came from the child interview and the demographic file and was limited to adolescents who were 12 years and older (9).

### Measures and variables

The first set of variables defined are those that are child report. These questions were asked of children aged 12–17 years. Previous research has documented the reliability of self-reported demographic data for adolescents (10, 11).

### Race/ethnicity

Race and ethnicity were asked in two questions. The first asked to identify their Hispanic ethnicity, asking participants to identify as: Spanish, Hispanic or Latino and then allowing them to select a subgroup (Mexican, Mexican American, Chicano, Puerto Rican, Cuban, or other Spanish?). Due to small sample sizes, we re-coded this variable into a dichotomous variable (Hispanic: yes/no).

Racial status was then asked, and participants were allowed up to three racial groups. The categories were: White, Black, American Indian, Alaskan Native, Asian, Native Hawaiian or Pacific Islander.

The final race/ethnicity variable used in this analysis combined the responses from the ethnicity and race questions. We re-coded their answers to their racial category (all three mentions) and the question about ethnicity to form the racial and ethnic groups used in this analysis. Due to small sample sizes, we aggregated to larger multi-racial groups based on many theories of multiracial identity and identification that demonstrate that in the United States, being multiracial and Black is a different experience than identifying as multiracial and presenting as White. In this conceptualization, Hispanic is treated as a racial group and therefore anyone who identifies as Hispanic and one or more racial group is considered multiracial. Black multiracial is any individual who identifies as more than one racial and/or ethnic group that includes Black (e.g., Black–Hispanic, Black–White, Black–Asian). White multiracial is classified as any individual who identifies as more than one race and/or ethnic group that includes White (except for Black-white which is categorized as Black multiracial). The final categories were: White-only (not Hispanic), Black-only (not Hispanic), Asian only (not Hispanic), White multiracial (including Hispanic) and Black multiracial (including Hispanic).

#### Religious services attendance

Primary caregivers are asked as part of the CDS to answer if their child has attended religious services in the last year.

#### Educational expectations

Primary caregivers are also asked what level of education they expect their child to reach. Their options ranged from grade 11 or less, graduate from high school, post-high school vocational training, some college, graduate from 2 year college with associate's degree, graduate from 4 year college, master's degree or teaching credential program and finally MD, law, PhD or other doctoral degree. We then re-categorized this variable into four categories as seen in Table 1.

**Table 1:** Sociodemographic characteristics across the racial and ethnic groups.

	**Overall sample**	**White only**	**White multiracial**	**Black only**	**Black multiracial**	**Asian only**	**Hispanic only**	**Others**	***P*** **-value**
	***N*** **(%)/M(SD)**	***N*** **(%)/M(SD)**	***N*** **(%) or M(SD)**	***N*** **(%) or M(SD)**	***N*** **(%) or M(SD)**	***N*** **(%) or M(SD)**	***N*** **(%) or M(SD)**	***N*** **(%) or M(SD)**	
***N*** **(%)**	1,094 (100%)	423 (55%)	83 (14.5%)	454 (14%)	58 (3%)	12 (2%)	45 (9%)	19 (2.5%)	
**Sex** ^*^									0.005
Male	411 (49%)	181 (52%)	35 (58%)	159 (49%)	17 (27%)	8 (71%)	6 (21%)	5 (38%)	
Female	421 (50%)	182 (48%)	26 (42%)	155 (50%)	27 (73%)	3 (29%)	22 (79%)	4 (62%)	
**Age**									0.4400
12–13	414 (33%)	151 (33%)	34 (33%)	163 (34%)	21 (40%)	3 (23%)	18 (32%)	8 (30%)	
14–15	383 (34%)	140 (32%)	30 (38%)	162 (33%)	21 (32%)	5 (37%)	15 (37%)	9 (66%)	
16–18	314 (32%)	132 (36%)	19 (28%)	129 (33%)	16 (27%)	4 (40%)	12 (31%)	2 (4.5)	
**Household income**	88,104 (5,379)	107,758 (9,430)	85,949 (7,408)	54,888 (3,334)	38,967 (5,411)	100,384 (20,027)	50,975 (5,290)	5,2563 (11,496)	< 0.000
**Urbanicity**									< 0.000
Urban	1074 (67%)	230 (56%)	55 (72%)	351 (77%)	51 (96%)	11 (91%)	36 (82%)	14 (77%)	
Suburban	167 (14%)	68 (15%)	14 (14%)	34 (9%)	3 (2%)	1 (9%)	8 (18%)	3 (17%)	
Rural	266 (20%)	124 (29%)	14 (14%)	69 (14%)	4 (2%)	0 (0%)	1 (0.6%)	2 (6%)	
**Attended religious services in last year**									0.9779
Yes	336 (33%)	140 (32%)	26 (35%)	124(32%)	18 (26%)	6 (49%)	16 (39%)	5 (22%)	
No	758 (67%)	280 (67%)	57 (65%)	323 (68%)	40 (74%)	6 (50%)	24 (61%)	13 (78%)	
**Parent expectation: child education**									0.4919
HS or lower	204 (14%)	53 (12%)	12 (14%)	117 (24%)	10 (11%)	2 (13%)	4 (9%)	2 (7%)	
Some college	56 (5%)	18 (4%)	8 (9%)	22 (5%)	2 (6%)	0 (0%)	2 (5%)	0 (0%)	
College graduate	680 (69%)	291 (71%)	53 (66%)	239 (62%)	38 (71%)	9 (78%)	27 (69%)	15 (83%)	
Masters or higher	134 (12%)	56 (13%)	9 (11%)	53 (9%)	8 (12%)	1 (9%)	6 (17%)	1 (10%)	

* Sex has a lower overall n because it was asked in a separate optional section of the survey and 266 respondents did not fill this optional section out.

### Psychosocial variables

#### Self-rated health

Participants are asked to rate their general health, “In general, would you say your health is excellent, very good, good, fair, or poor”?

#### Children's depression inventory short form

Ten questions are asked as part of the children's depression inventory scale (12), asking about their feelings over the last 2 weeks. These individual items were then combined to make a scale that is used to assess severity of depression-related symptoms. In order to improve accuracy and protect privacy, adolescents were told to read these questions themselves and answer with a code that corresponded to the statement that best described their feelings. This scale was treated continuously for our analyses, with higher scores meaning more depressive symptoms.

#### Rosenberg self-esteem scale

Five questions were asked as part of the Rosenberg self-esteem scale (13). These individual items were then combined to make a scale used to assess self-esteem. This scale was treated continuously for our analyses, with higher scores meaning higher self-esteem.

### Peer influences

Participants were asked to answer how many of their friends engaged in a number of “positive” activities (participating in community groups, volunteer groups, thinking school is important, etc.) and “negative” behaviors (skip classes, engagement in violence).

#### Peer problems scale

The peer problems scale addresses to what extent adolescents get along with their peers. Five items are taken from the “strengths and difficulties questionnaire” (14) to evaluate children's problems with peers in the last 6 months. A scale was included in the PSID dataset for these items that added them together and created a composite score (with a higher score representing more peer problems).

#### Peer victimization and bullying

The peer victimization and bullying scale (15) consisted of four items that were pulled from Kochender and Ladd. The PSID dataset included an aggregate scale by adding the responses to these variables together and creating a composite score (with a higher score signifying higher rates of peer victimization and bullying).

## Analysis

### Survey weights

The CDS 2014 provided weights to allow researchers to generalize results to the national population of children and their caregivers. Because the focus of this paper is the adolescents, we used the weight that was developed for research questions that were looking at adolescents as the subgroup of interest.

Descriptive statistics were used to describe the population and to explore family and parent variables to gain a better understanding of multiracial families in the United States using this national sample—these variables included racial/ethnicity identification, geographic context, household income and sex. We explored psychosocial outcomes such as depressive symptoms and self-esteem by racial and ethnic status and differences in peer treatment and peer group behaviors by racial and ethnic status.

## Results

Table 1 summarizes the sociodemographic characteristics of the overall sample and separately by race and ethnic group (self-report by the child) for this nationally representative sample. The weighted percent of White multiracial adolescents was 14.5% and Black multiracial adolescents was 3%. Household income and geographic location differed significantly between the racial and ethnic identification groups. White single-race adolescents were from households that report the highest average household income ($107,758) and adolescents who identified as Black single-race reported an average household income of $54,888. Households that included White multiracial adolescents fell in-between with an average household income of $85,949. The lowest reported average household income was among Black multiracial—$38,967. Multiracial adolescents were more likely to live in urban areas. Fifty-six percent of households with White single-race adolescents reported living in an urban area, whereas for White multiracial this number is 72% and Black multiracial 96%. There were no significant differences between racial and ethnic groups for attending religious services or for parental expectation of child education.

Table 2A presents weighted averages for the three health and psychosocial scales. There were similar self-reported health scores for multiracial adolescents in comparison to their single-race peers. Black multiracial youth scored lower on the CDI (3.2) than their single-race Black peers (9.4). Multiracial adolescents scored the same or higher than their single race peers on the Rosenberg self-esteem scale.

**Table 2A:** Adolescent report of psychosocial variable: averages by race and ethnicity.

	**Overall sample**	**White only**	**White multiracial**	**Black only**	**Black multiracial**	**Asian only**	**Hispanic only**	**Other**
Self-rated health	2.1 (0.04)^*^	2.0 (0.04)	2.1 (0.12)	2.1 (0.09)	2.2 (0.21)	2.7 (0.37)	2.5 (0.17)	2.6 (0.20)
Children's depression inventory short-form	4.6 (0.58)	4.3 (0.71)	3.2 (1.3)	9.4 (2.7)	3.2 (1.3)	7.5 (4.1)	2.8 (0.69)	2.1 (0.66)
Rosenberg self-esteem scale	16.7 (0.10)	16.7 (0.13)	16.8 (0.30)	17.4 (0.21)	17.6 (0.50)	15.4 (0.61)	15.5 (0.46)	16.5 (0.59)

* Mean (SD) reported.

Table 2B summarizes the survey linear regressions performed to test for differences between single-race and multiracial adolescents while controlling for income. The models were done separately for White multiracial adolescents and Black multiracial adolescents with each being compared to their single-race peers. The only significant difference that was found was for depressive symptoms: Black multiracial adolescents had significantly lower scores (b = −5.9, *p* = 0.04) on the depressive symptoms index (even when controlling for income) when compared to Black single-race.

**Table 2B:** Adolescent report of psychosocial variables: regression models comparing multiracial and single-race adolescents^*^.

	**Children's depression inventory**		**Rosenberg self-esteem**	
	**Coefficient**	***p*** **-value**	**Coefficient**	***p*** **-value**
**Racial and ethnic group**				
White only	Ref	Ref	Ref	Ref
White multiracial	−1.19	0.412	0.19	0.561
Black only	Ref	Ref	Ref	Ref
Black multiracial	−5.90	0.042	0.16	0.619

* Controlling for household income.

Tables 3A, B summarize findings about peer problems and bullying for multiracial and single-race adolescents. No significant differences were found between racial groups for the peer problems scale. White multiracial adolescents reported higher average scores (more negative treatment from peers) than their White single-race peers; in contrast, however, lower rates of victimization were reported for Black multiracial peers relative to their Black single-race peers. Table 3B presents the regression models for these outcomes separately for White multiracial adolescents and Black multiracial adolescents. The only significant finding was that White multiracial adolescents were at increased risk for peer victimization and bullying in comparison to their single-race White peers (b = 0.95, *p* = 0.05), while controlling for income.

**Table 3A:** Adolescent report of peer problems and bullying: averages by race and ethnicity.

	**Overall sample**	**White only**	**White multiracial**	**Black only**	**Black multiracial**	**Asian only**	**Hispanic only**	**Other**
Peer problems scale	3.0 (0.43)^*^	2.0 (0.09)	4.6 (2.0)	2.7 (0.50)	1.9 (0.26)	2.3 (0.56)	7.3 (3.4)	2.1 (0.30)
Peer victimization and bullying	3.5 (0.14)	3.0 (0.16)	3.9 (0.45)	4.2 (0.32)	3.6 (0.70)	1.8 (0.42)	4.9 (0.63)	6.2 (1.1)

* Mean (SD) reported.

**Table 3B:** Adolescent report of peer problems and bullying: regression models comparing multiracial and single-race adolescents^*^.

	**Peer problems scale**		**Peer victimization and bullying**	
	**Coefficient**	***p*** **-value**	**Coefficient**	***p*** **-value**
**Racial and ethnic group**				
White only	Ref	Ref	Ref	Ref
White multiracial	2.35	0.238	0.95	0.048
Black only	Ref	Ref	Ref	Ref
Black multiracial	−0.74	0.185	−0.91	0.227

* Controlled for income.

Analyses examined peer group behaviors for multiracial adolescents and young adults in comparison to their single-race peers. Survey regressions comparing multiracial and single-race adolescents uncovered few significant differences between groups, even after adjusting for household income. The only significant difference was for White multiracial adolescents who had a higher mean score than their single-race White peers for having peers who think school is important (b = 0.47, *p* < 0.001), a finding that remains even after controlling for income (b = 0.49, *p* < 0.001).

**Figure 1:**
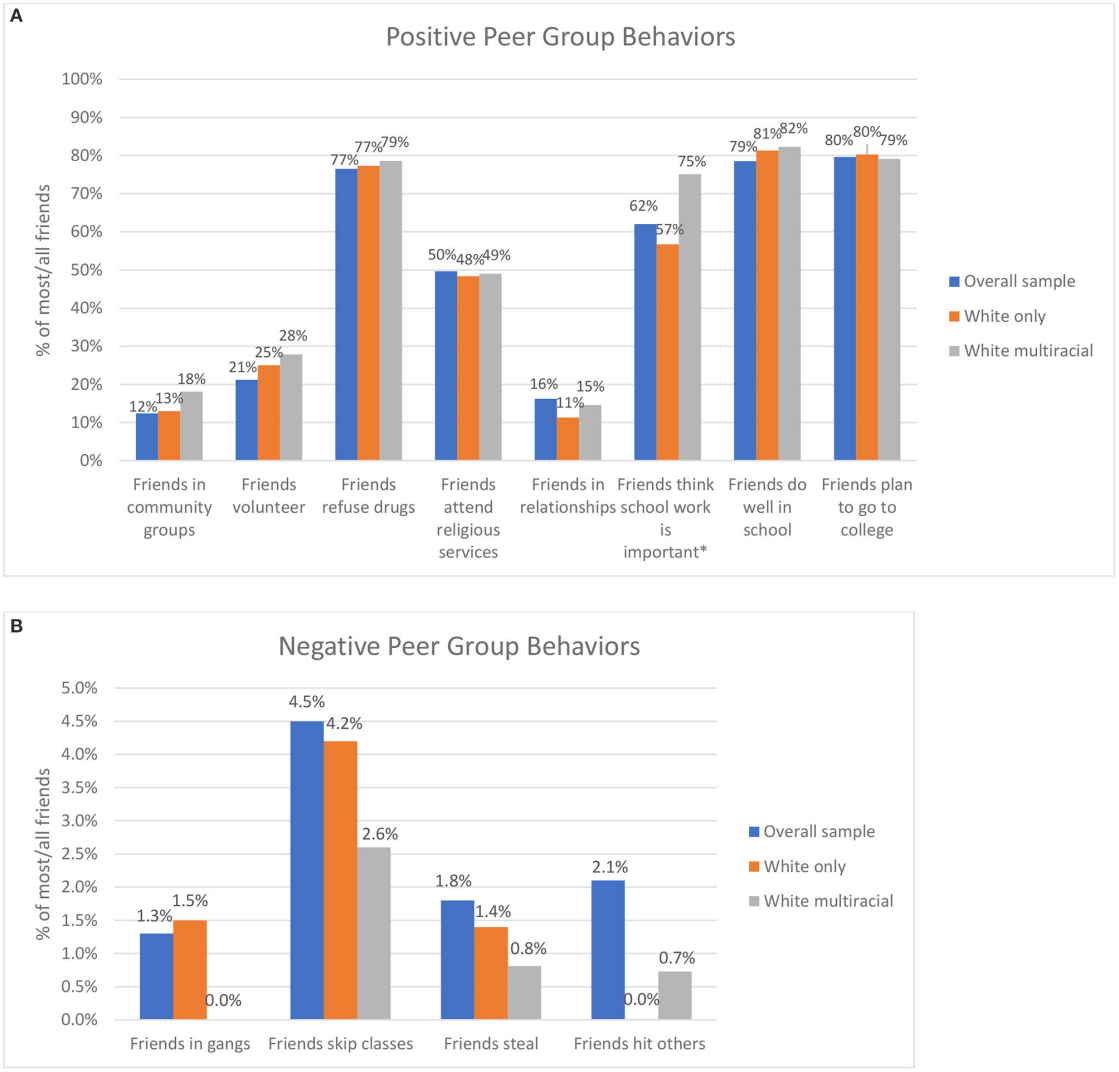
**(A)** Positive peer influences for white multiracial adolescents. **(B)** Negative peer influences for white multiracial adolescents.

**Figure 2:**
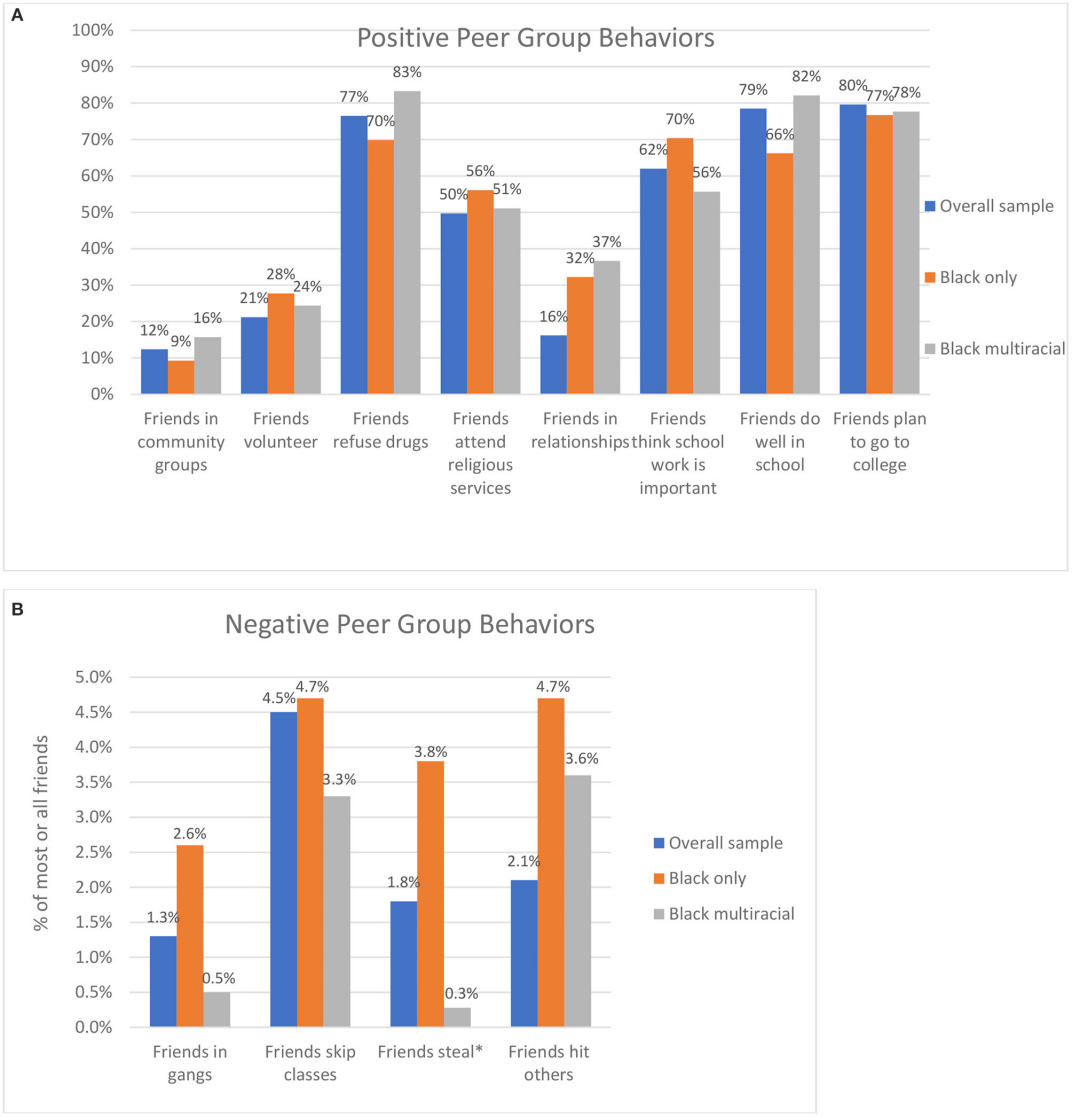
**(A)** Positive peer influences for black multiracial adolescents. **(B)** Negative peer influences for black multiracial adolescents.

Figures 1A, B, 2A, B show the percentage of adolescents by racial/ethnic group who report that all or most of their friends engage in a series of positive peer group behaviors and negative peer group behaviors. Inspection of the Figures reveals the similarities in peer groups for these different racial and ethnic identifications. Figure 1A demonstrate that when compared to White single-race peers, White multiracial peers report similar percentages of friends who engage in positive behaviors. Figure 1B shows that White multiracial adolescents report similar or lower raw percentages of friends who report engaging in these negative peer behaviors.

A similar pattern emerges in Figures 2A, B which present the same positive and negative peer behaviors for Black single-race and Black multiracial identifying adolescents. In almost all cases Black multiracial adolescents report higher or about the same percentage of most/all of their friends engaging in positive behaviors such as refusing drugs, doing well in school, and planning to go to college. Also, in all four negative peer group behaviors, Black multiracial adolescents reported lower percentages of friends engaging in these behaviors when compared to Black single-race identifying adolescents, although these may not be significant differences, they demonstrate the pattern that multiracial adolescents do not have peers that engage in significantly more risk behaviors, and in fact might be trending toward having less risky peer groups.

## Discussion

### Demographic patterns

A complex picture emerges when looking at patterns across multiracial and single-race adolescents and their families. When examining demographic characteristics, it appears that there is some level of socioeconomic disadvantage among multiracial families. Households with White multiracial adolescents report household incomes that are lower than households with single-race White adolescents, and the same relationship appears for households with Black multiracial adolescents reporting lower household incomes than households with their single-race Black peers. When exploring urbanicity in the context of these findings regarding income disparity, a similar pattern emerges. A higher percentage of White multiracial adolescents report living in urban areas in comparison to their White peers and a higher percentage of Black multiracial adolescents report living in an urban area compared to their Black peers. These findings around income and urbanicity should be further studied, as it is important to understand why these patterns might be emerging. The finding around urban areas is particularly interesting—is this due to the concentration of poverty in urban areas, or is it due to greater acceptance of inter-racial and inter-ethnic families in urban areas, or most likely, is it reflection of a confluence of these different factors?

### Psychosocial outcomes

Complex patterns emerge when examining the psychosocial and peer treatment variables presented in this analysis for multiracial adolescents and young adults and their single-race peers. The findings regarding depressive symptoms and peer bullying point to signs of different relationships between Black multiracial adolescents and their single-race Black peers and White multiracial adolescents and their single-race White peers. There is not a perfect continuum where multiracial adolescents are always between both White and Black single-races, however the patterns that do emerge point toward White multiracial adolescents reporting worse outcomes than their White single-race peers, but Black multiracial adolescents reporting better outcomes than their Black single-race peers. For example, the findings around depressive symptoms demonstrated that Black multiracial adolescents reported significantly lower depressive symptoms when compared to their single-race Black peers. When exploring peer bullying and treatment, White multiracial adolescents reported higher bullying scores than their White-single-race peers.

A particularly notable finding was that Black multiracial and Black single-race youth reported the highest self-esteem scale scores. This finding about self-esteem, especially in the context of the findings regarding depressive symptoms, demonstrates an impressive amount of resilience that should not be overlooked. Instead of applying a risk-based approach oft used when considering adolescents (particularly adolescents of color), the power in this ability to maintain high levels of self-esteem should be harnessed and supported by those working with and advocating for adolescents. The dearth of research and discussion of these psychosocial strengths in youth of color have been documented previously, especially in how this framing improperly shapes how professionals who work with these populations view their mental health and in particular their strengths (16).

Previous health outcomes literature that examined multiracial adolescents has often taken a risk-based approach that has assumed stress and anxiety must be the mechanisms for negative health outcomes for multiracial adolescents and young adults (3, 4). More recent studies, such as the one by Weller and colleagues demonstrate that it may not be the multiracial identity itself that is “risky” but that some experiences known to influence depression in adolescents such as household dysfunction may bring unique challenges to multiracial adolescents when forming their racial and ethnic identities (6). The finding that in many ways Black multiracial adolescents are reporting more positive outcomes than their single-race Black peers, but White multiracial adolescents are reporting more negative outcomes than their White single-race peers fits into the historical context of race relations in the United States. The racial order in the United States has always relied upon and exploited a Black-White divide and has privileged Whiteness. Black multiracial adolescents may be conferred some of this privilege, buffering them from some of the treatment that leads to poor psychosocial outcomes often reported by their Black single-race peers. This pattern also emerges when looking at White multiracial adolescents who report worse outcomes than their White single-race peers as their multiracial identification may be preventing them from receiving the full privilege bestowed on their only White peers. These findings around multiracial identification are elucidating the idea that privilege is still conferred based on Whiteness in the United States. However, it is critical that research moving forward does not examine multiracial identity in a vacuum—and that the influences of intersecting social identities including sex, socioeconomic status, sexuality and others are considered when examining axes of privilege and oppression.

### Peer influences

The third major aim of the paper was to examine relationships and influences of peers for multiracial identifying adolescents. Some previous research has claimed that due to feeling a need to try harder to fit in, multiracial adolescents may be more susceptible to peer pressure (4). In this nationally representative sample of youth, positive peer behaviors in peer networks are as high or higher in multiracial adolescents and negative peer behaviors are often lower for multiracial adolescents when compared to their single-race peers. This suggests that in many ways Black multiracial peers had more positive and less negative behaviors when compared to their single-race Black peers. This finding demonstrates a possible buffer that exists for multiracial youth—that not being identified as “fully” or “exclusively” in a minority category may confer some level of privilege to these youth. It may also point to multiracial adolescents having more diverse racial and ethnic peer groups than their single-race peers—something that this paper did not have the data to test but should be explored in future research. Another potential difference in terms of influences for multiracial adolescents that should be further explored is that of their parents and extended families. It is possible that multiracial families have more diverse networks and therefore peer networks which may influence behaviors and outcomes for multiracial youth. It is critical to understand peer groups and influences on adolescents, as we know that during this period of development, peer networks are highly influential on preventing adolescent risk behavior involvement (17). Further research, quantitative and qualitative, should be done to explore this idea of a continuum of treatment and perception for multiracial youth and to talk with youth who identify as multiracial about their peer group decisions and influences.

### Limitations

The major limitation of this study was the relatively small sample sizes, a limitation that characterizes much of the existing research on multiracial data. Due to this small sample size, we were unable to examine more nuanced groups of multiracial identification and needed to aggregate to Black multiracial and White multiracial. Another limitation is that the race/ethnicity data collected from the parent and adolescent were not collected at the same time as the adolescent report comes from the data in 2014 and the parent report comes from the birth history file. Therefore, we don't know if the parent has also changed how they identify their child over time. An additional limitation is that many current studies use the CES-D and not the children's depression short form to look at depressive symptoms among children. However, because this was a secondary data analysis we were not able to influence which measures were chosen.

### Implications

As more research is conducted that aims to examine multiracial adolescents and young adults in the United States, it is important that nationally representative samples are used to demonstrate what this sample looks like descriptively. These data also demonstrate that a risk-based approach is not appropriate when studying multiracial adolescents, and that their resiliency should be harnessed and supported. Future research should continue to create and utilize nuanced multiracial groups and to test mechanisms of mental health and peer networks before assuming risk. Research should also continue to elucidate the ways in which privilege is conferred to different racial and ethnic identifications. Many people theorized that the rise of multiracial populations would begin to erode the color line—but it might instead be reifying it. It will be critical for future research to examine if multiracial populations are given privilege and treated differently than minority single-race peers, and if that difference in treatment deepens the historical Black-White divide in the United States.

## Data availability statement

The data analyzed in this study is subject to the following licenses/restrictions: You must apply for data through the Panel Study of Income Dynamics. Requests to access these datasets should be directed to https://psidonline.isr.umich.edu.

## Ethics statement

The studies involving human participants were reviewed and approved by Columbia University Irving Medical Center IRB. Written informed consent to participate in this study was provided by the participants' legal guardian/next of kin.

## Author contributions

SG conceptualized the paper, performed the analyses, and drafted the manuscript. JS, CN, MC, AA-L, SA, and DH advised SG on the conceptualization of the paper and reviewed and edited all drafts of the manuscript. All authors contributed to the article and approved the submitted version.

## References

[1] Jones N Marks R Ramirez R. 2020 Census Illuminates Racial Ethnic Composition of the Country. Census.gov. (2022). Available online at: https://www.census.gov/library/stories/2021/08/improved-race-ethnicity-measures-reveal-united-states-population-much-more-multiracial.html (accessed October 3, 2022).

[2] Rockquemore KA Brunsma DL Delgado DJ. Racing to theory or retheorizing race? Understanding the struggle to build a multiracial identity theory. J. Soc. Issues. (2009) 65:13–34.

[3] Udry JR Li RM Hendrickson-Smith J. Health and Behavior Risks of Adolescents with Multiracial Identity. Am J Public Health. (2003) 93:1865–70. 10.2105/AJPH.93.11.1865 14600054 PMC1448064

[4] Choi Y He M Herrenkohl TI Catalano RF Toumbourou JW. Multiple identification and risks: Examination of peer factors across multiracial and single-race youth. J Youth Adolesc. (2012) 41:847–62. 10.1007/s10964-012-9750-2 22395776 PMC3372672

[5] Fisher S Reynolds JL Hsu WW Barnes J Tyler K. Examining multiracial youth in context: Ethnic identity development and mental health outcomes. J Youth Adolesc. (2014) 43:1688–99. 10.1007/s10964-014-0163-2 25100614

[6] Weller BE Conrad JK Wilburn VG Ramamonjiarivelo Z Gladden J. Adverse childhood experiences and mental health conditions among multiracial adolescents. Ethnicity Health. (2022) 27:1088–102. 10.1080/13557858.2020.1869187 33472407

[7] Echols L Ivanich J Graham S. Multiracial in middle school: the influence of classmates and friends on changes in racial self-identification. Child Dev. (2018) 89:2070–80. 10.1111/cdev.13000 29178469 PMC6105562

[8] Grilo SA Santelli JS Nathanson CA Catallozzi M Abraido-Lanza A Adelman S. Social and structural influences on multiracial identification and health: a public health mandate to precisely measure, theorize, and better understand multiracial populations. J Racial Ethnic Health Disparities. (2022) 10:427–45. 10.1007/s40615-022-01234-5 35192180

[9] Mainieri T. Panel Study of Income Dynamics, Child Development Supplement 2014: User Guide. Michigan: Institute for Social Research, University of Michigan (2017).

[10] Klein JD Graff CA Santelli JS Hedberg VA Allan MJ Elster AB. Developing quality measures for adolescent care: validity of adolescents' self-reported receipt of preventive services. Health Serv Res. (1999) 34:391–404. 10199683 PMC1089009

[11] Santelli J Klein J Graff C Allan M Elster A. Reliability in adolescent reporting of clinician counseling, health care use, and health behaviors. Med Care. (2002) 40:26–37. 10.1097/00005650-200201000-00005 11748424

[12] Overholser JC Brinkman DC Lehnert KL Ricciardi AM. Children's depression rating scale-revised: development of a short form. J Clin Child Psychol. (1995) 24:443–52. 10.1207/s15374424jccp2404_8

[13] Rosenberg M. Conceiving the Self . New York: Basic Books (1986).

[14] Goodman R. The strengths and difficulties questionnaire: a research note. J Child Psychol Psychiatry. (1997) 38:581–6. 10.1111/j.1469-7610.1997.tb01545.x 9255702

[15] Kochenderfer BJ Ladd GW. Peer victimization: cause or consequence of school maladjustment? Child Develop. (1996) 67:1305–17. 10.1111/j.1467-8624.1996.tb01797.x 8890485

[16] Vera E Thakral C Gonzales R Morgan M Conner W Caskey E. Subjective well-being in urban adolescents of color. Cultural Diversity Ethnic Minority Psychol. (2008) 14:224. 10.1037/1099-9809.14.3.224 18624587

[17] Maxwell KA. Friends: The role of peer influence across adolescent risk behaviors. J Youth Adolesc. (2002) 31:267–77. 10.1023/A:1015493316865

